# BMP-2 (and partially GDF-5) coating significantly accelerates and augments bone formation close to hydroxyapatite/tricalcium-phosphate/brushite implant cylinders for tibial bone defects in senile, osteopenic sheep

**DOI:** 10.1007/s10856-023-06734-2

**Published:** 2023-06-28

**Authors:** André Sachse, Ines Hasenbein, Peter Hortschansky, Klaus D. Schmuck, Stefan Maenz, Bernhard Illerhaus, Peter Kuehmstedt, Roland Ramm, René Huber, Elke Kunisch, Victoria Horbert, Francesca Gunnella, Andreas Roth, Harald Schubert, Raimund W. Kinne

**Affiliations:** 1 Experimental Rheumatology Unit, Orthopedic Professorship, Jena University Hospital, Waldkliniken Eisenberg GmbH, Eisenberg, Germany; 2 Orthopedic Professorship, Jena University Hospital, Waldkliniken Eisenberg GmbH, Eisenberg, Germany; 3 Leibniz-Institute for Natural Products Research and Infection Biology—Hans-Knoell-Institute, Jena, Germany; 4 Johnson & Johnson Medical GmbH, DePuy Synthes, Norderstedt, Germany; 5 Chair of Materials Science, Otto Schott Institute of Materials Research, Friedrich Schiller University Jena, Jena, Germany; 6 Federal Institute for Materials Research and Testing (BAM), Berlin, Germany; 7 Fraunhofer Institute for Applied Optics and Precision Engineering IOF, Jena, Germany; 8 Institute of Clinical Chemistry, Hannover Medical School, Hannover, Germany; 9 Bereich Endoprothetik/Orthopädie, Klinik für Orthopädie, Unfallchirurgie und Plastische Chirurgie, Uniklinik Leipzig AöR, Leipzig, Germany; 10 Institute of Laboratory Animal Sciences and Welfare, Jena University Hospital, Jena, Germany

## Abstract

Bilateral defects (diameter 8 mm) in the medial tibial head of senile, osteopenic female sheep (*n* = 48; 9.63 ± 0.10 years; mean ± SEM) were treated with hydroxyapatite (HA)/beta-tricalcium phosphate (β-TCP)/dicalcium phosphate dihydrate (DCPD; brushite) cylinders coated with BMP-2 (25 or 250 micrograms) or growth differentiation factor (GDF)-5 (125 or 1250 micrograms; left side); cylinders without BMP served as controls (right side). Three, 6, and 9 months post-operation (*n* = 6 each group), bone structure and formation were analyzed in vivo by X-ray and ex vivo by osteodensitometry, histomorphometry, and micro-computed tomography (micro-CT) at 3 and 9 months. Semi-quantitative X-ray evaluation showed significantly increasing bone densities around all implant cylinders over time. High-dose BMP-2-coated cylinders (3 and 9 months) and low-dose GDF-5-coated cylinders (3 and 6 months) demonstrated significantly higher densities than controls (dose-dependent for BMP-2 at 3 months). This was confirmed by osteodensitometry at 9 months for high-dose BMP-2-coated cylinders (and selected GDF-5 groups), and was again dose-dependent for BMP-2. Osteoinduction by BMP-2 was most pronounced in the adjacent bone marrow (dynamic histomorphometry/micro-CT). BMP-2 (and partially GDF-5) significantly increased the bone formation in the vicinity of HA/TCP/DCPD cylinders used to fill tibial bone defects in senile osteopenic sheep and may be suitable for surgical therapy of critical size, non-load-bearing bone defects in cases of failed tibial head fracture or defect healing.

**Graphical Abstract:**

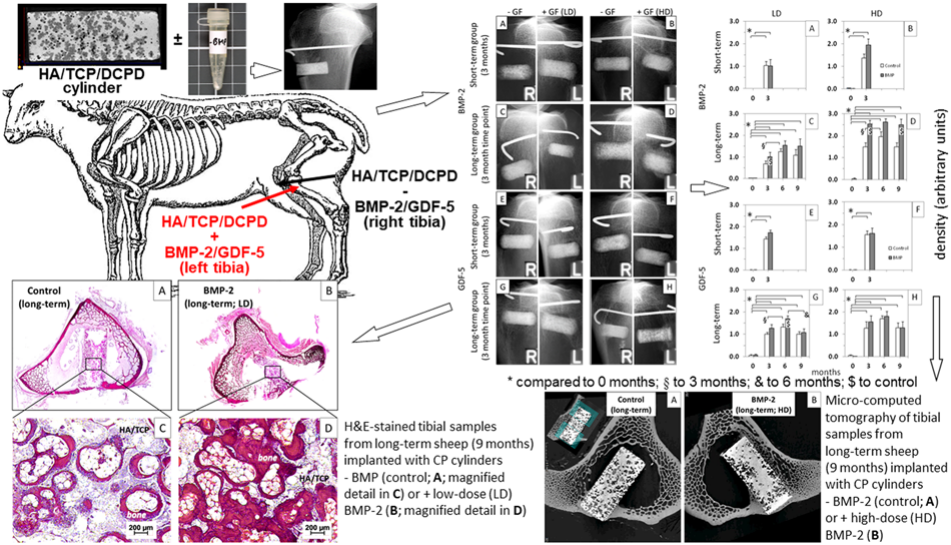

## Introduction

Bone replacement materials are essential for the therapy of critical size, non-load-bearing bone defects in a number of clinical diagnoses, e.g., pseudarthrosis, bone cysts or tumors, osteomyelitis or avascular bone necrosis, tibial head fractures, and opening-wedge osteotomy [1–6]. For this purpose, allografts and/or xenogenic/synthetic bone replacement materials are used, since the supply of the gold standard autologous bone is limited (e.g., due to a large critical size of the primary defect or to insufficient bone quality at the harvest site; [3, 7, 8]). This includes degradable or non-degradable polymers (e.g., collagen, or polymethylmethacrylate), calcium phosphates (CP) such as hydroxyapatite (HA), tricalcium-phosphate (TCP), or brushite (DCPD), and xenogenic bone preparations (e.g., Surgibone; [8]). However, in particular large bone defects in old patients with high-energy trauma and comorbidities may result in “osteogenic insufficiency” and may thus require additional activation of the bone graft, for example by bone morphogenetic proteins (BMP; [9]). This has stimulated the development of osteoinductive BMPs in conjunction with CP replacement materials in cases such as spine surgery, maxillofacial applications, and ankle arthrodesis [8, 10, 11].

Bone morphogenetic proteins mediate the osteoinductive activity of demineralized bone for ectopic bone formation in adult animals [12, 13] and are pivotal for bone development, osteogenic cell differentiation, and fracture healing [14–16]. BMP-2 is a member of the superfamily of transforming growth factor-β (TGF-β) proteins, know to activate so called type I and type II TGF-β receptors [17–19].

Several therapeutically efficacious BMPs have been tested in animal models of osteopenia [20–26]. Systemic administration of recombinant human (rh)BMP-6 increased bone volume and restored the microarchitecture and quality of bone in aged, ovariectomized rats [27]; also, local injection of rhBMP-7 into osteopenic ovine vertebrae improved bone mechanical strength and histomorphometric parameters [28], and the dental implantation of β-TCP with rhGD5 (also called rhBMP14) in dogs enhanced the healing of peri-implant defects [29, 30].

BMP-2 triggers osteogenesis through autocrine and paracrine mechanisms, as shown by elegant in vitro and ex vivo studies [14, 31]. In addition, BMP-2 improves the in vivo parameters of bone structure or bone formation in mice [20], rats [21], rabbits [32], goats [22], and sheep [23, 25] and represents a very promising molecule for cervical [33] or lumbar [34] spine fusions, with further applications for alveolar/dental surgery [35]. This was the basis for the marketing of several clinical products containing rhBMP-2 or rhBMP-7 for fracture therapy or spinal fusion [16, 19].

In addition, growth differentiation factor (GDF-)5 is a pivotal factor for bone formation and regeneration, as shown by delayed fracture healing of femur fractures and impaired early matrix deposition and callus formation in knock-out mice [17, 36, 37], as well as a relative lack of GDF-5 expression in human non-union vs. normally healing fractures [38]. This is further supported by: (1) the equality of GDF-5-coated β-TCP and autologous bone grafts for human maxillary sinus augmentation [39, 40]; (2) the superiority of GDF-5-coated vs. non-coated PLGA, β–TCP, or collagen 1 for alveolar bone augmentation in dogs [29], healing of titanium dental implants in dogs [30], and healing of osteochondral defects in mini-pigs [41], respectively; (3) faster healing of a rat critical size femur defect with a superagonistic GDF-5 mutant [42]; and iv) significant enhancement of the bone formation induced by PLGA fiber-reinforced calcium phosphate cement (CPC) in sheep lumbar osteopenia by GDF-5 or its mutant BB1 [24, 26].

Currently, there is only very limited information on the use of BMP-containing bone replacement materials in the tibial head [43]. Thus, the current study was undertaken to investigate the therapy of tibial bone defects in a large animal sheep model with BMP-2 or GDF-5-coated HA/TCP/DCPD cylinders (brushite-enriched Conduit^TM^ R). Senile osteopenic sheep were used, which show markedly diminished bone structure and formation, substantially augmented bone erosion, and a high similarity to human pathology [44, 45]. This model fulfils FDA recommendations for a non-rodent, large animal model with intracortical bone remodeling, and was chosen to reflect age-dependent challenges of defects in the tibial head, e.g., rarification of anatomically defined bone columns, increase in the proportion of fatty bone marrow, and reduced bone healing capacity due to limited stem cell recruitment and delayed vessel ingrowth [45–48].

## Materials and methods

### Preparation of BMP-2 or GDF-5-coated HA/TCP/DCPD (CP) cylinders

**Fig. 1:**
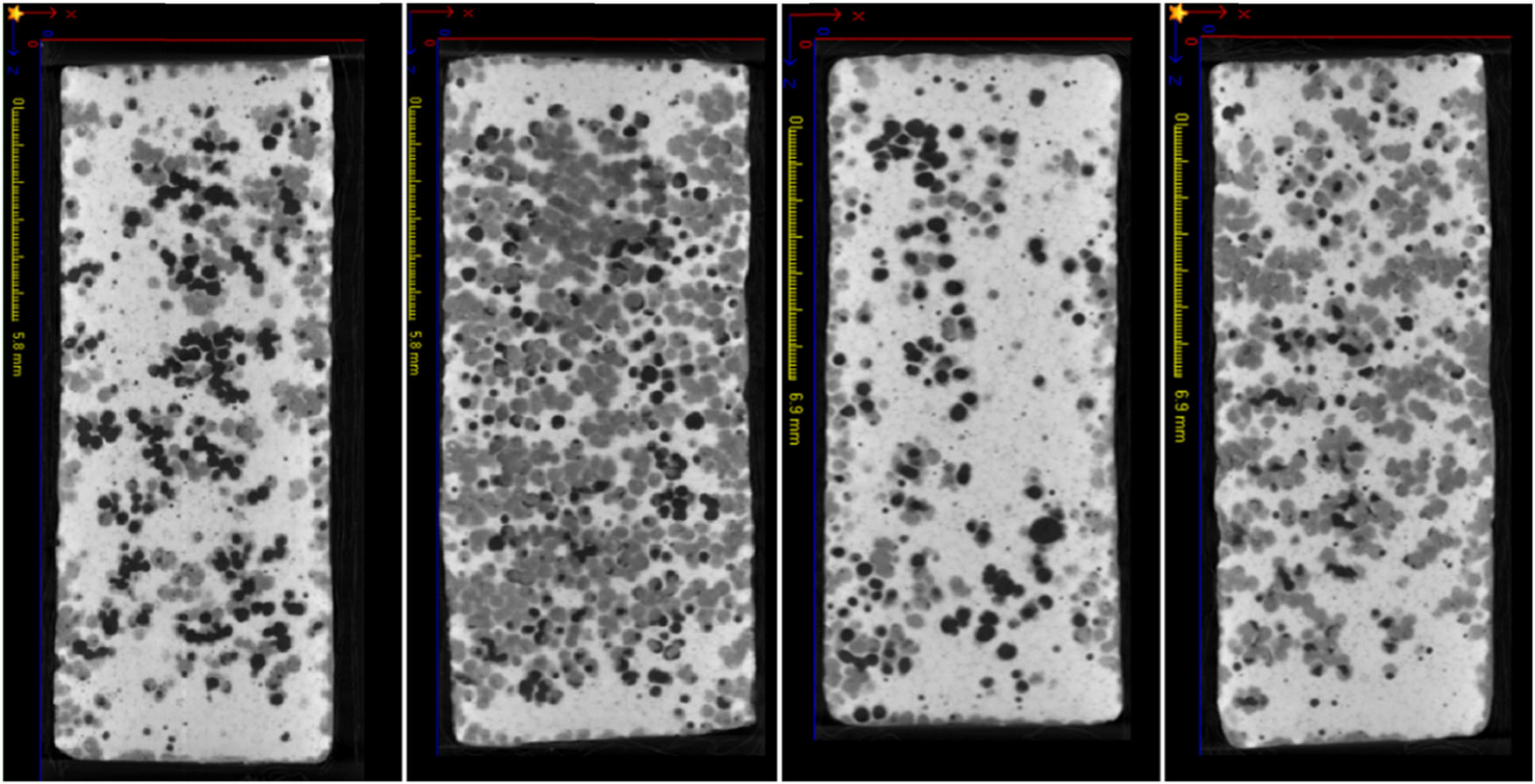
Variable morphology of four representative implanted HA/TCP/DCPD cylinders. The morphology of the cylinders was characterized by micro-computed tomography (micro-CT), resulting in areas of high density (white; HA/TCP), medium density DCPD (gray), and pores (black; for details see section “Micro-computed tomography (micro-CT”)

Bioresorbable, synthetic bone replacement cylinders consisting of sintered 75% HA and 25% β-TCP were used as implants (diameter 8 mm; length 20 mm; tradename Conduit^TM^ R; Kasios S.A.S., L’Union, France). The material showed an initial porosity of 60–80% with interconnecting pores of a diameter between 200–500 μm. To increase the mechanical strength of the implants, the pores were filled with dicalcium phosphate dihydrate (DCPD; brushite) by immersion with a saturated DCPD solution (Table 1). This resulted in cylinders with variable morphology and a compressive strength of 10 MPa, an excellent accessibility of the interior of the cylinder for bone-forming cells, and final relative proportions of HA/β-TCP, DCPD, and pores of 47.6%, 43.4%, and 9.0%, respectively (*n* = 5; Fig. 1).

**Table 1:** Observation periods of senile osteopenic sheep receiving BMP-coated cylinders (*n* = 6 in each group)

Growth factor	Duration	Dose	BMP-2/GDF-5 dosage
BMP-2	Short-term (3 months)	Low	25 µg BMP-2
		high	250 µg BMP-2
	Long-term (9 months)	low	25 µg BMP-2
		high	250 µg BMP-2
GDF-5	Short-term (3 months)	low	125 µg GDF-5
		high	1250 µg GDF-5
	Long-term (9 months)	low	125 µg GDF-5
		high	1250 µg GDF-5

Calcium phosphate (CP) cylinders: Hydroxyapatite (75%)/β-tricalcium phosphate (25%)/dicalcium phosphate dihydrate (pore filling); for each group, controls were the contralateral tibia of the same sheep, receiving CP cylinders without BMP-coating

Finally, the cylinders were coated with recombinant human BMP-2 (rhBMP-2; 25 and 250 µg) or rhGDF-5 (125 and 1250 µg) by application of the BMP-containing solutions on the cylinders and subsequent sterile drying in circulating air at room temperature (Table 1; [49]). In brief, non-glycosylated BMP-2 was produced in *E. coli* using patented procedures (patent DE 199 44 626 A1; [49] and references therein) also commonly used for clinical trials in humans, GDF-5 by analogous procedures. The present dosages were chosen on the basis of BMP doses successfully applied in previous experiments with BMP-coated hydroxyapatite-titanium tibia implants [45]. Bioactivity of BMP-2 and GDF-5 was confirmed using the pre-myoblastic C2C12 cell line [45] and a biosensor test system with an immobilized ectodomain of the BMP receptor IA (ALK-3; data not shown). Calculating the surface area required for single layer binding of BMP-2 or GDF-5, the loading protein should have been completely immobilized on the surface of the CP cylinders. Although the release kinetics of the loaded protein were not formally analyzed, an almost complete cumulative release of the surface-coated BMPs from the CP cylinders in serum within 14 days can be assumed on the basis of previous results from HA-particles ([49] and references therein).

### Surgical procedure

Senile, osteopenic female sheep were used (48 Merino sheeps; 9–10 years old, except for one sheep with an age of 6 years; 45–82 kg body weight, 60.4 ± 1.18 kg, mean ± SEM; *n* = 6 for each experimental and control group [44, 45]). The experimental sheep were raised in a flock under controlled standard rearing conditions and fed standard animal chow for the whole pre-experimental period on the basis of an initial long-term contract with a sheep farm (Schäferei Hänsch, Jena; Germany) under regular monitoring by the research group and under the supervision of the responsible local authority (Thüringer Landesamt für Verbraucherschutz, Abteilung gesundheitlicher und technischer Verbraucherschutz, Bad Langensalza, Germany).

With the exception of one group comparison not showing any significant differences for any other parameter in the data analyses, there were no significant differences among the body weights of the different experimental groups, thus making an experimental influence of the body weight highly unlikely. In comparison to young sheep (2–4 years), the lumbar vertebral body L3 of the senile sheep showed only marginally diminished bone mineral density, but significantly decreased structural and bone formation parameters, as well as significantly increased bone erosion, suggesting that senile sheep may represent a suitable model of senile osteopenia [44]. An overall reduction of X-ray absorption and trabecular organization, a lack of a defined border toward the marrow cavity, and a thinning of the cortical bone was also observed in the tibia of senile sheep [45]. Power analyses (G*Power [26]) for differences between the control and verum BMP groups for the X-ray values at 3 or 9 months confirmed that sample sizes between 4 and 7 sheep were sufficient to detect differences with an alpha error probability of 0.05, a power (1-β error probability) of 0.80, and an effect size between 1.83 and 2.47. Permission for the animal experiments was obtained from the governmental commission for Animal Welfare, Free State of Thuringia, Germany (registration number: 02-21/05). All experiments were conducted in accordance with the National Institutes of Health (NIH) Guidelines for the Care and Use of Laboratory Animals.

**Fig. 2:**
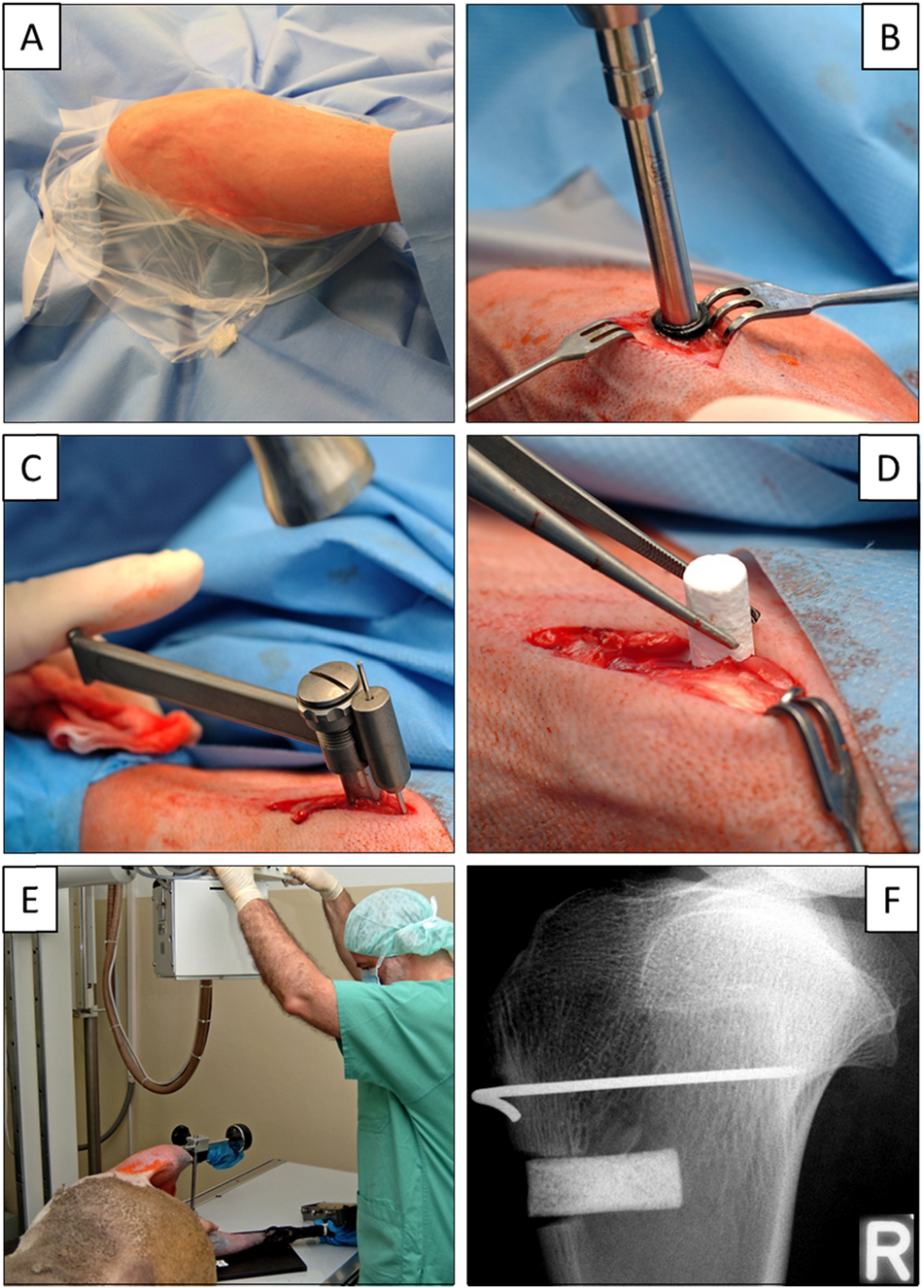
Surgical technique. **A** Stifle joint operation situs following disinfection and sterile covering of upper and lower limb by a surgical drape; **B** Skin incision of 40 mm; identification of the appropriate defect position on the medial surface of the right tibial head at the horizontal level of the tibial tuberosity (half distance between tuberosity and posterior edge of the tibia; center of the visible medial surface); defect generation with an orthograde orientation using a drill (8 mm trephine; Stryker); **C**, **D** Defect placement of the implant using Kirschner guide wire and surgical adapter; **E**, **F** X-ray imaging to exclude either implant protrusion from the tibial surface or sinking into the depth of the tibia

**Fig. 3:**
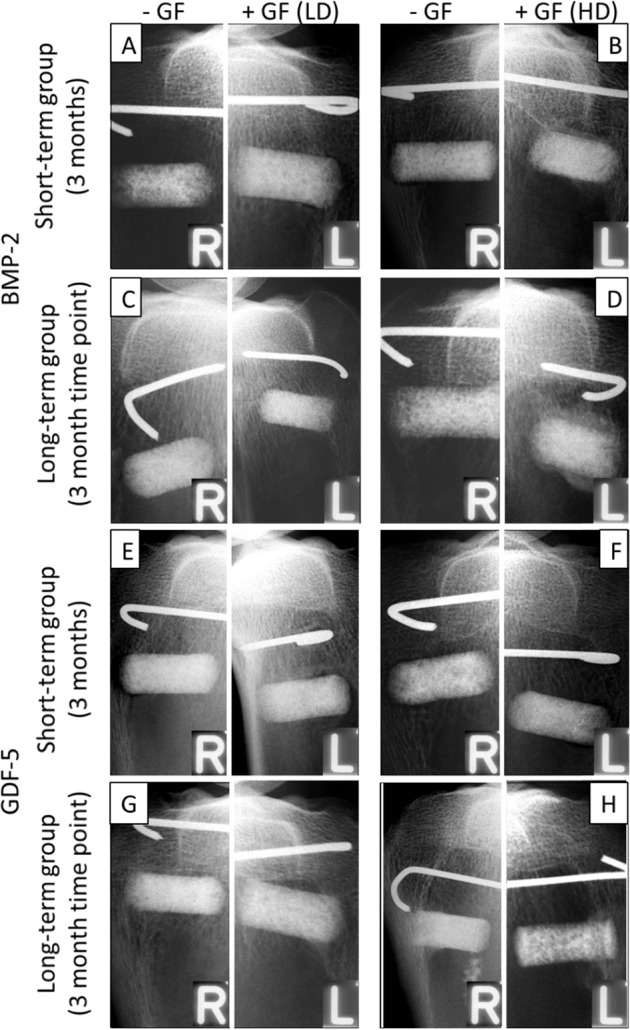
X-ray analysis of the tibial head with the respective implant cylinders without growth factor (“−GF = control”) and with growth factor (“+GF”) either in low-dose (LD) or high-dose (HD); “BMP-2” (**A**–**D**) or “GDF-5” (**E**–**H**) at the 3 months’ time point in the short-term (**A**, **B**, **E**, **F**) and at 9 months in the long-term groups (**C**, **D**, **G**, **H**). Bone formation in the LD and HD BMP-2 and GDF-5 groups was consistently higher than in the respective controls without BMPs

**Fig. 4:**
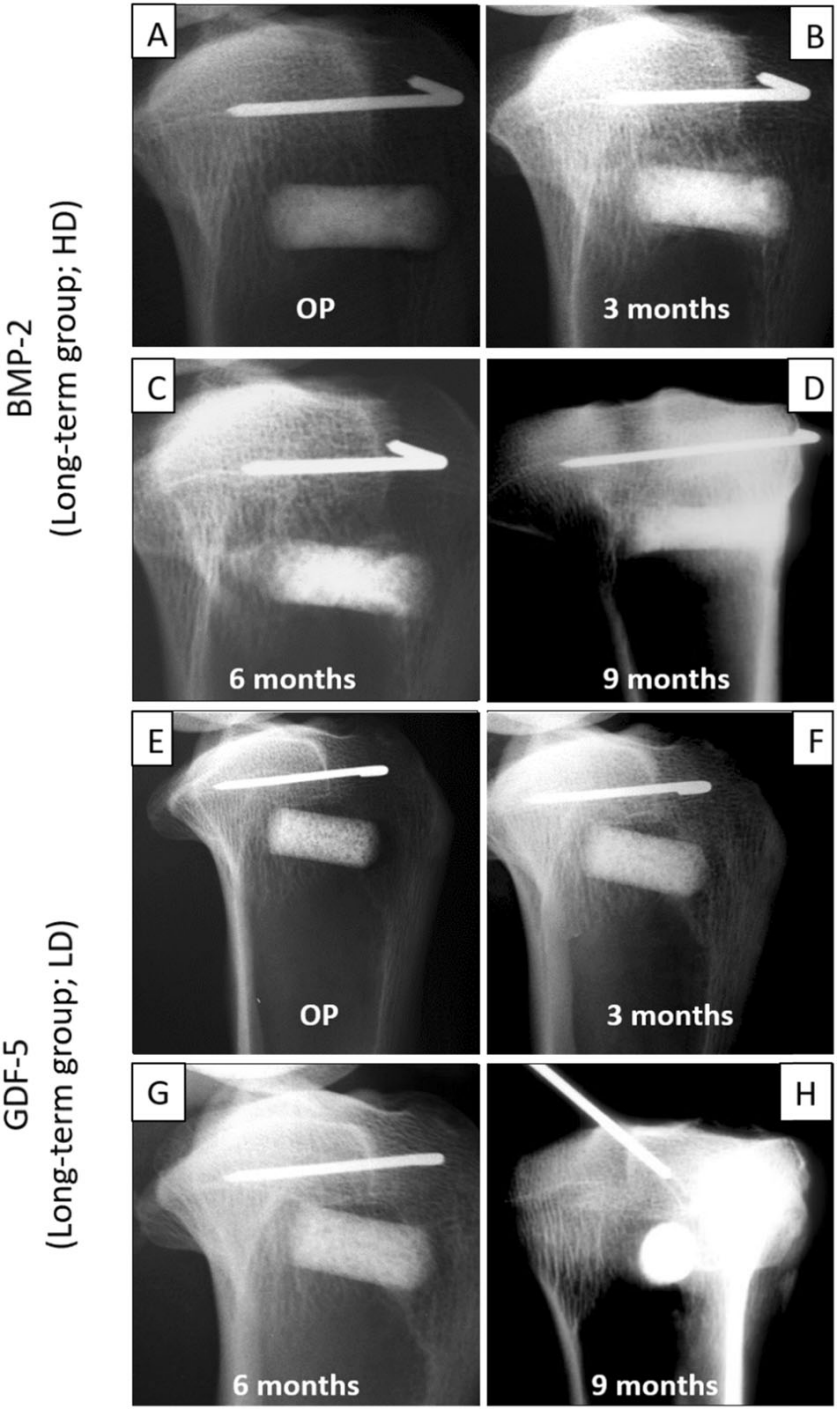
Time course of the effects of the BMP-coated implant cylinders in the tibial head concerning the “best-matching dosages of BMP-2” (high-dose [HD]; 250 µg (**A**–**D**)) and “GDF-5” (low-dose [LD]; 125 µg (**E**–**H**)). X-rays were taken immediately after surgical insertion (OP) and at subsequent 3 month intervals

After shaving and disinfecting the operation region of the sheep under sedation (Ketamin™; Zoetis Deutschland GmbH, Berlin, Germany; 100 mg/ml, 12 ml; 17 mg/kg body weight), surgery was performed using a ventral access to the tibia (Fig. 2A, B). Briefly, anesthesia was induced by intravenous injection of 2 mg/kg Propofol (Fresenius Kabi Deutschland GmbH, Bad Homburg, Germany) and then maintained by inhalation of isoflurane™ at 1.5–1.8 (v/v; AbbVie, Ludwigshafen, Germany). The sheep were optimally positioned on their back under X-ray control. After a skin incision of 40 mm, the appropriate position for the bone defect was identified on the medial surface of the “right tibial head” at the horizontal level of the tuberositas tibiae (i.e., half distance between the tuberositas and the posterior edge of the tibia to bypass the load-bearing ventral and medial-posterior corticalis columns; center of the visible medial surface; Fig. 2A, B). A bone defect with an orthograde orientation to the medial surface was generated using a drill (8 mm diameter; Stryker Leibinger GmbH, Freiburg, Germany; Fig. 2B), and the “control implant cylinder without BMP” was inserted into the defect using a surgical adapter and a metal guide wire (Kirschner wire; Fig. 2C, D), which was randomly located around the cylinder, but did not show any clear positional effects on the bone healing around the CP cylinders (compare with Figs. 3 and 4 below). Particular attention was paid to avoid any protrusion of the cylinder from the tibial surface or a sinking into the depth of the tibia (Fig. 2E, F), the latter often avoided by generating contact of the cylinder with the endost of the opposite corticalis (compare with Figs. 3 and 4 below). The same procedure was repeated for the “contralateral left tibia (cylinder with BMP)” of the same animal using separate skin incisions and access channels. Bone defects in the right and left tibia were filled with CP cylinders without (control) and with BMP-2 or GDF-5, respectively.

After surgery, sheep were kept in separate sheds for 1–2 weeks and then transferred to long-care paddocks for 3 months (short-term) or 9 months (long-term). Except for the tracking period, the short-term and long-term groups did not differ from each other. Post-operative medication consisted of antibiotics (Ampicillin-sodium, twice daily 10 mg/kg body weight for 4 days, Ratiopharm GmbH, Ulm, Germany; Enrofloxacin, once daily 2.5 mg/kg for 4 days, Bayer, Leverkusen, Germany) and antiphlogistics (Metamizol-Sodium, twice daily 2 mg/kg for 4 days, Wirtschaftsgenossenschaft Deutscher Tierärzte—WdT, Garbsen, Germany; Carprofen, twice daily 2 mg/kg for 2.5 days, Pfizer Animal Health,Berlin, Germany).

In the long-term group, bone structure and formation were also analyzed in vivo by X-ray (see below) after 3 and 6 months following short sedation of the sheep with Propofol (0.2 mg/kg/h). After 3 or 9 months, the sheep (*n* = 24, each) were sacrificed using i.v. injection of overdosed barbiturate (Pentobarbital™, Essex Pharma GmbH, Munich, Germany), followed by application of magnesium sulfate (MgSO_4_, Dr. Paul Lohmann GmbH, Emmerthal, Germany). To avoid seasonal effects on bone structure, formation, and erosion, the animals were sacrificed with a balanced distribution in the four seasons spring, summer, autumn, and winter. The tibia was removed using an oscillating bone saw, analyzed by X-ray (Optimus 50; Philips GmbH; Hamburg, Germany) and kept frozen until further use. A semi-quantitative visual examination of the bone density around the CP cylinder was performed in all X-rays using a scoring system with 0 = no bone formation; 1 = weak bone formation; 2 = moderate bone formation; 3 = strong bone formation. This evaluation system, which is very close to the procedure routinely used for X-ray examination in the clinical setting, was newly established in the present study and validated via blinded, separate evaluation of the X-rays by two operators (A.S.; R.W.K) unaware of the group characteristics of the sample, which yielded highly congruent results.

### Digital osteodensitometry

Osteodensitometry was executed using a software-guided digital bone density measuring instrument (DEXA QDR 4500 Elite™; Hologic, Waltham, MA, USA), using a rectangular region of interest (size 9 × 23 mm). High density areas of the inserted CP cylinder were excluded from quantification.

### Histology and histomorphometry

After cutting the tibia samples into two parts directly along the axis of the CP cylinder, evaluations were performed using two different types of sections: (1) decalcified paraffin sections stained by hematoxylin-eosin [50]; or (2) plastic-embedded sections obtained by fixation in acetone and dehydration in ascending alcohol series without demineralization. The latter samples were embedded in Technovit 9100 in line with the instructions of the supplier (Heraeus Kulzer, Wehrheim, Germany; [51]). Sections were then cut and ground to a thickness of ~7 μm.

### Dynamic histomorphometrical measurements

To calculate dynamic histomorphometric indices, sheep were given intramuscular injections of oxytetracycline (OTC; 20 mg/kg body weight and 10 mg lidocaine) 1 and 2 days after surgery and subsequently at 3, 6, and 9 months. Short-term groups received their 3rd injection 10 days before sacrifice, while the long-term group animals received a total of 5 injections (including one at 10 days before sacrifice). Oxytetracycline is incorporated into newly formed osteoid and thus “dynamically” marks bone formation within ~10 days after application (compare with images in [49, 52]). Dynamic histomorphometrical analyses were performed in plastic-embedded sections using a fluorescence microscope (Axiovert 200 M^™^, Carl Zeiss, Microimaging GmbH, Oberkochen, Germany), 20-fold magnification, a Zeiss PlanNeoFluar-objective (excitation wavelength of 390 nm; emission wavelength of 512 nm) with an AxioCam color camera (12 V DC, 0.7 A) and the respective software (AxioVision 3.8, Carl Zeiss). The Vidost tetra software was used for image analysis (Videoplan, 1991), with the operator being unaware of the group characteristics of the sample. All measured and calculated parameters are based on published nomenclature [49, 53–56].

### Micro-computed tomography (micro-CT)

For the acquisition of 3-D images, an X-RAY WorX 225 kV tube micro-CT system (X-RAY WorX GmbH, Garbsen, Germany) with a flat panel detector was used (PerkinElmer 1621; CsJ as scintillator; 2048 × 2048 pixel; PerkinElmer, Waltham, USA) as previously published [49].

Quantitative analysis of the micro-CT data was carried out using the 3-D software VGSTUDIO MAX 2.2 (Volume Graphics GmbH, Heidelberg, Germany) and applying cylinders with a radius of 4.0, 4.5, 5.0, 5.5, and 6.0 mm (see insert in Fig. 10A). The position and longitudinal axis of the implant cylinders were defined on the basis of the co-implanted Kirschner guide wire. Bone volume (BV) and total volume (TV) were separately determined by global threshold determination in the drill channel (maximal diameter of 8.0 mm) and adjacent cylinder segments (principle of onion shell) in the corticalis and the bone marrow. For this purpose, the respective maxima of gray values for soft tissue, bone tissue/DCPD, and CP cylinder were determined (compare with Fig. 1) and the means between the maxima were used as a threshold for the volume determination of the individual components. The bone volume/total volume (BV/TV) was subsequently calculated.

### Statistics

The data were expressed as means ± standard errors of the mean for the different groups. The Wilcoxon test was used to analyze the results of paired samples (samples from the same sheep) for statistically significant differences. Differences between values for 3 and 9 months, BMP-2 and GDF-5, or the 3-month time point in the respective short-term and long-term groups were analyzed using the Mann–Whitney *U* test. If applicable, multi-group tests (Friedmann and Kruskal–Wallis tests, respectively) were first performed, and only parameters showing significant differences among groups in the multi-group tests were further analyzed in bilateral post hoc tests for differences between different time points, control and BMP groups, and different BMPs. For all tests, the level of significance was set at *p* ≤ 0.05. All statistical tests were performed using the Sigmaplot software release 26.0 (Systat Software Inc., Chicago, USA).

## Results

### X-ray analysis

At the 3-month time point, the bone formation in the low-dose and high-dose verum groups for BMP-2 (Fig. 3A–D) and GDF-5 (Fig. 3E–H) was consistently higher than in the respective control groups without BMPs. Increasing bone formation until the 3-month time point with a plateau thereafter was observed when looking at the time course in the high-dose, long-term BMP-2 group (125 µg; Fig. 4A–D) and the low-dose, long-term GDF-5 group (250 µg; Fig. 4E–H).

**Fig. 5:**
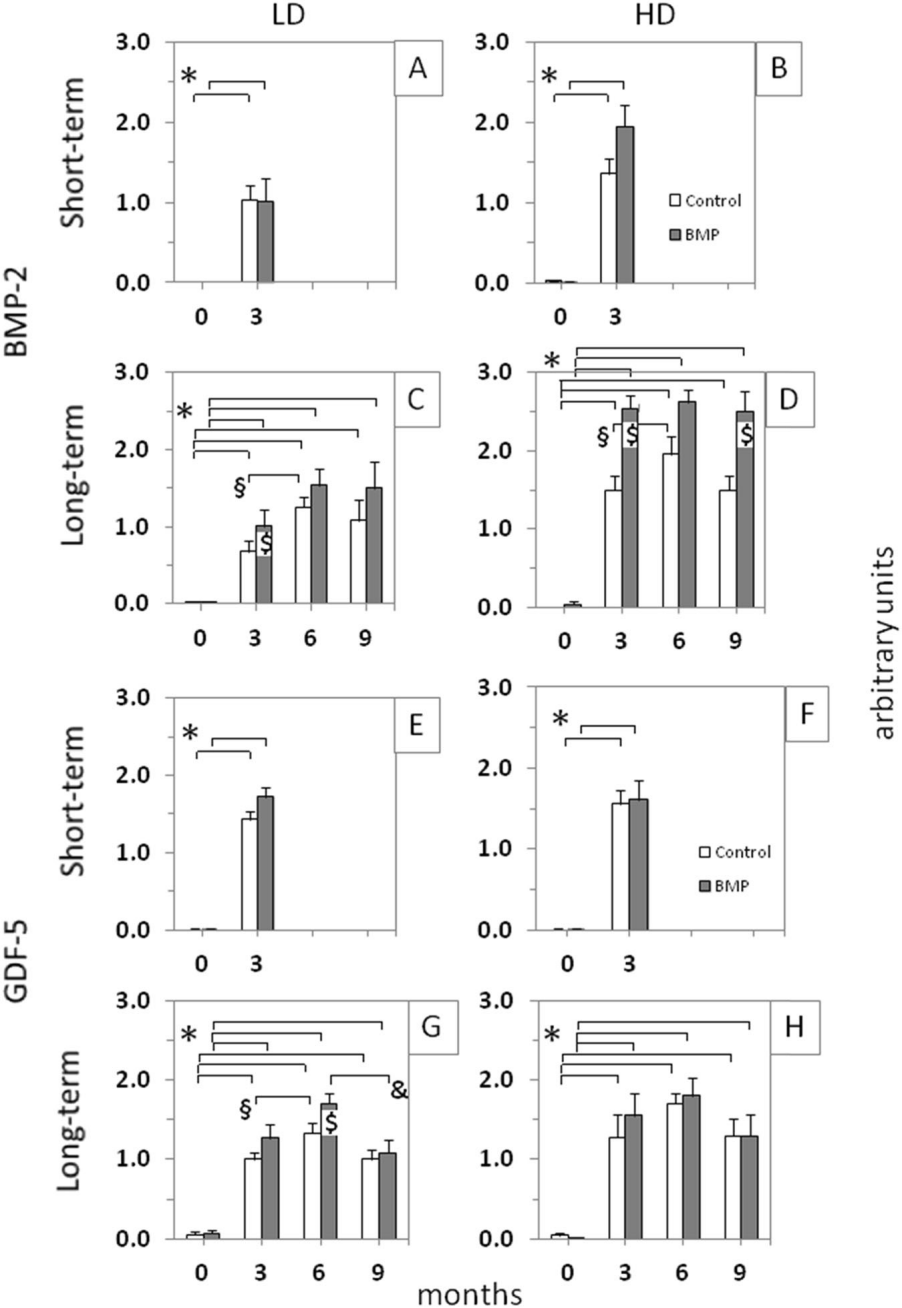
Semi-quantitative evaluation of the X-ray analyses (arbitrary units) immediately after surgical implant insertion (0 months) and at 3-month intervals in the short-term and long-term control or contralateral low-dose (LD) and high-dose (HD) BMP-2 (**A**–**D**) and GDF-5 groups (**E**–**H**; *n* = 6 for all groups); *p ≤ 0.05 vs. 0 months; ^§^*p* ≤ 0.05 vs. 3 months; ^&^*p* ≤ 0.05 vs. 6 months; ^$^*p* ≤ 0.05 vs. control

These results were confirmed by semi-quantitative evaluation of the X-rays, showing a significant increase of the bone formation at 3 months in both control and verum short-term groups of BMP-2 and GDF-5 (low-dose and high-dose; Fig. 5A, B, E, F). In the long-term, low-dose and high-dose BMP-2 and GDF-5 groups, these effects were extended until the 9-month time point (Fig. 5C, D, G, H). Whereas the BMP-2 and GDF-5 groups reached their plateau of maximal bone formation already at the early 3-month time point, the control groups needed a further significant increase from 3 to 6 months to reach this plateau and thereafter remained constant or even decreased (Fig. 5C, D, G, H). In selected long-term BMP-2 (low-dose, 3 months; high-dose, 3 and 9 months) and GDF-5 groups (low-dose, 6 months), the verum groups induced significantly higher bone formation than the control groups (Fig. 5C, D, G). When comparing the results for control or verum BMP at the 3-month time point between the respective short-term and long-term groups, only 1 comparison yielded a significant difference (low-dose GDF-5 verum; short-term vs. long-term; *p* = 0.036; Fig. 5E, G; significance not shown), indicating the high reproducibility of the results in the different experimental groups. The reasons for this single, possibly spurious significant difference are presently unclear and may include biological and/or experimental variability, in our view not questioning the validity of the present study.

**Fig. 6:**
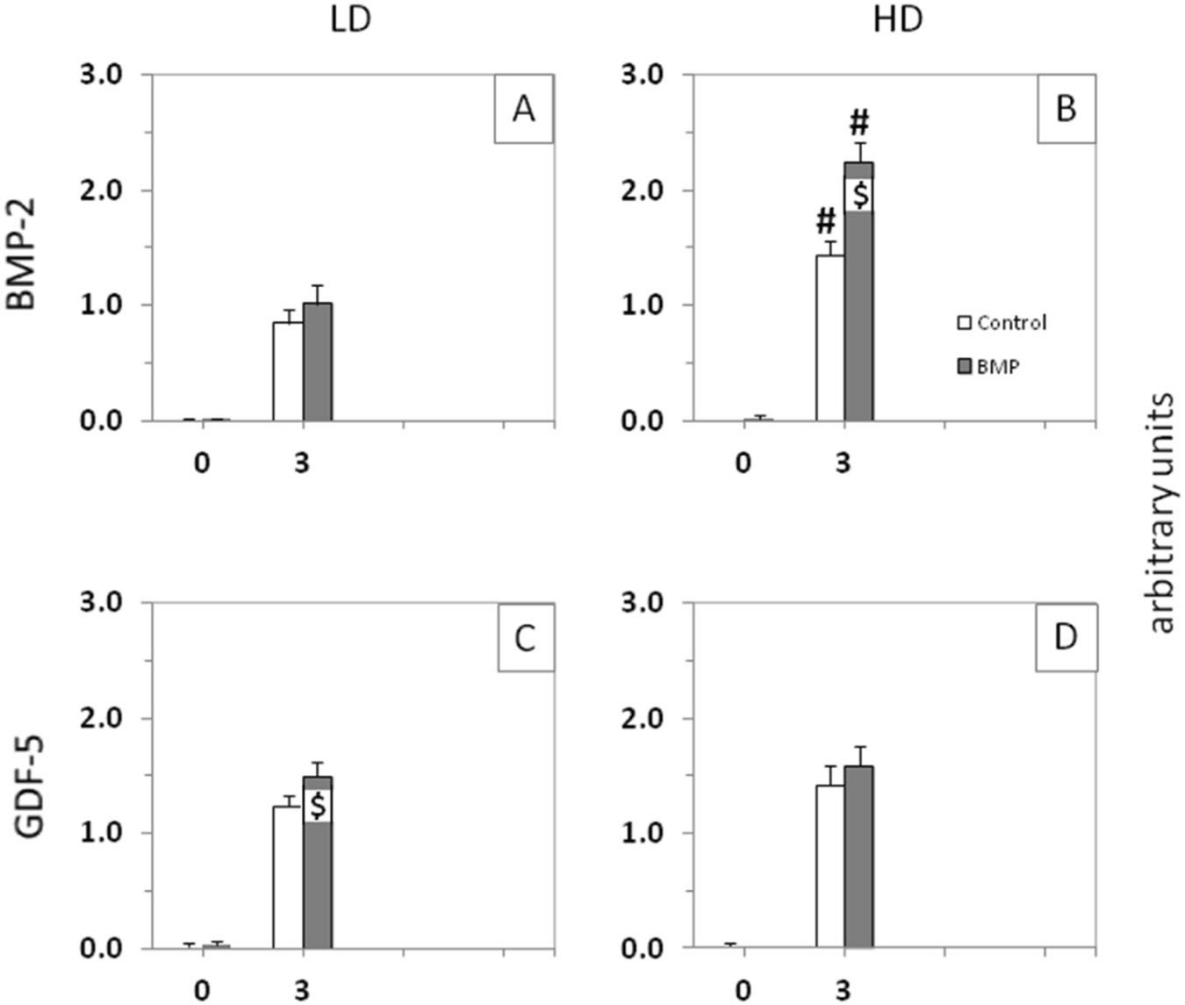
Semi-quantitative evaluation of the X-ray analyses (arbitrary units) immediately after surgical implant insertion (0 months) and at 3 months in the pooled short-term and long-term control or contralateral low-dose (LD) or high-dose (HD) BMP-2 (**A**, **B**) and GDF-5 groups (**C**, **D**; *n* = 12 each; white bars: control, gray bars: BMP, i.e., either BMP-2 or GDF-5); ^$^*p* ≤ 0.05 vs. control; ^#^*p* ≤ 0.05 vs. LD; for reasons of clarity, significant differences between different time points are only indicated in Fig. 5

**Fig. 7:**
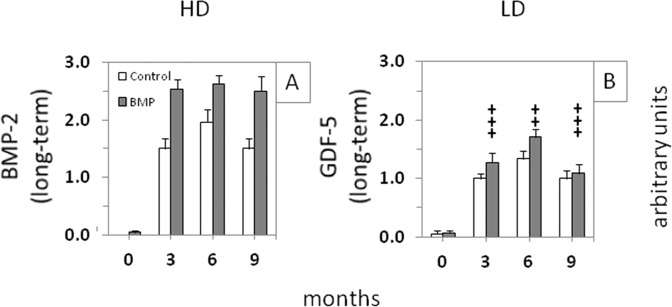
Semi-quantitative evaluation of the X-ray analyses (arbitrary units) immediately after surgical implant insertion (0 months) and at 3-month intervals throughout the whole time course in the long-term control or “best-matching dosages of BMP-2” (high-dose [HD]; 250 µg (**A**)) or “GDF-5” (low-dose [LD]; 125 µg (**B**)); ^+++^*p* ≤ 0.005, ^++^*p* ≤ 0.01 vs. high-dose BMP-2; for reasons of clarity, significant differences among different time points or between control and verum are only indicated in Fig. 5

When pooling the 3-month data for the short-term and long-term groups (*n* = 12 each), high-dose BMP-2 induced significantly higher bone formation than low-dose BMP-2 (Fig. 6B). When directly comparing the two different BMPs (long-term groups), the high-dose BMP-2 group (250 µg) was significantly more potent for the bone induction than the low-dose GDF-5 group (125 µg) at all time points (Fig. 7A, B).

### Osteodensitometry

**Fig. 8:**
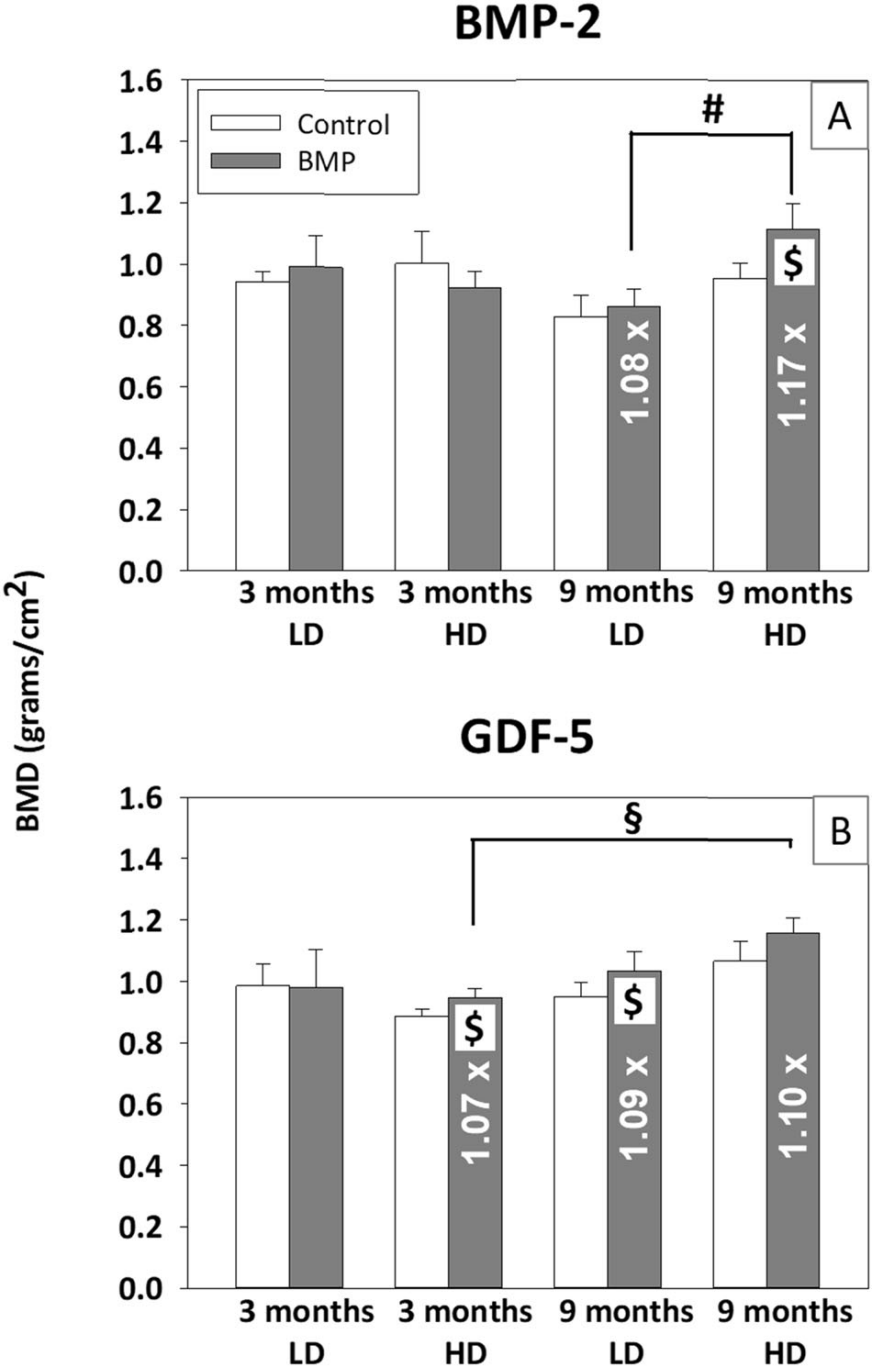
Bone mineral density (BMP; grams/cm^2^; osteodensitometry) at 3 and 9 months after surgical implantation in the short-term and long-term control or low-dose (LD) and high-dose (HD) BMP-2 (**A**) and GDF-5 groups (**B**); ^#^*p* ≤ 0.05 vs. LD; ^§^*p* ≤ 0.05 vs. 3 months; ^$^*p* ≤ 0.05 vs. control

In most of the experimental groups, the verum groups showed a numerically or significantly higher bone mineral density (BMD) than the control groups (*p* ≤ 0.05 vs. control for the 9-month, high-dose BMP-2 group, as well as the 3 month, high-dose and 9 month, low-dose GDF-5 groups; max. 1.17-fold; Fig. 8A, B). In addition, the BMD was dose-dependently higher in the 9 month, high-dose BMP-2 than the respective low-dose BMP-2 group (Fig. 8A) and time-dependently higher in the 9 month, high-dose GDF-5 compared to the respective 3-month GDF-5 group (Fig. 8B). For the long-term BMD, there were no significant differences between the high-dose BMP-2 (250 µg) and the low-dose GDF-5 group (125 µg; Fig. 8).

### Dynamic histomorphometrical measurements

**Fig. 9:**
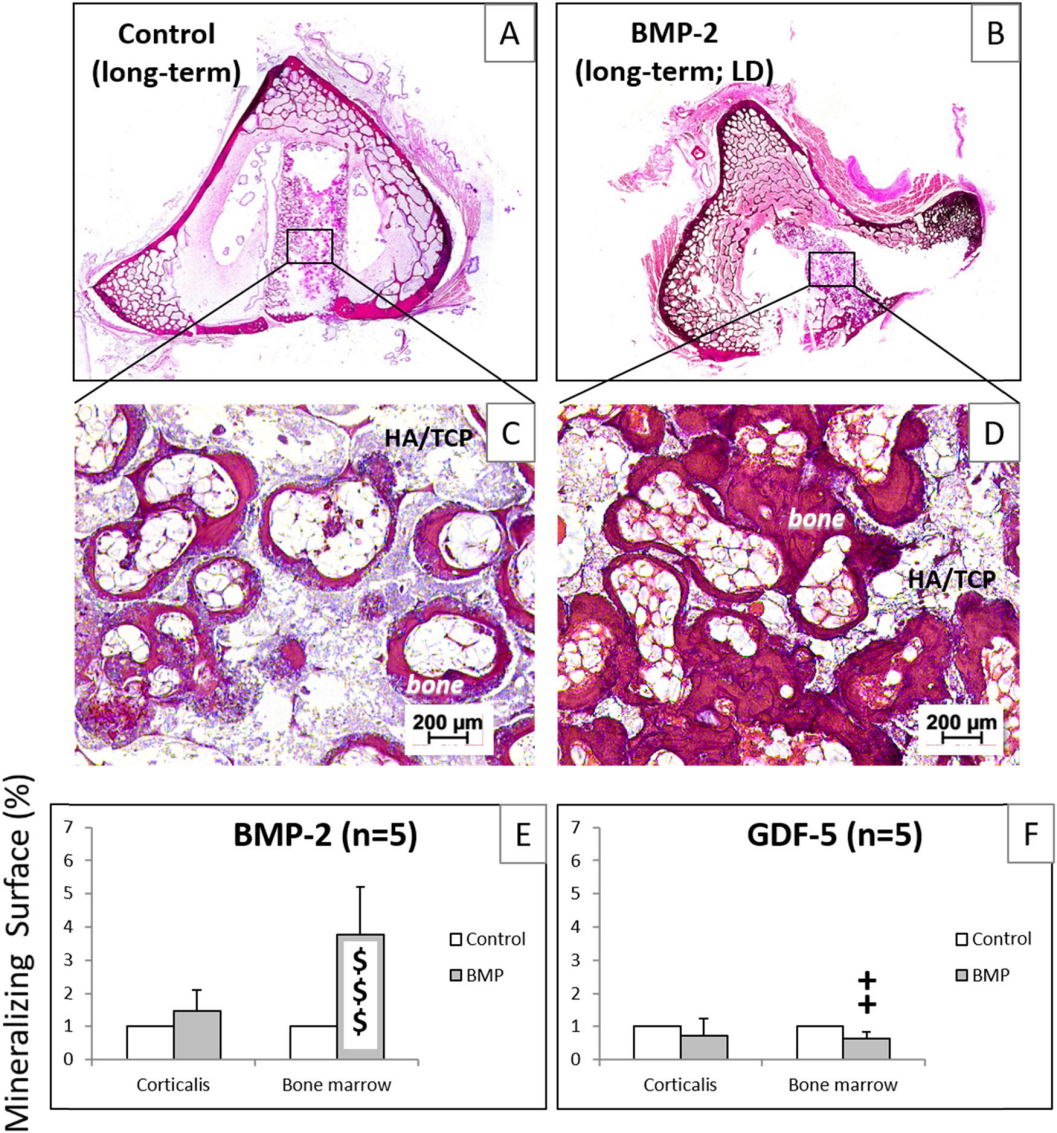
Hematoxylin-eosin stained paraffin sections of tibial samples from long-term sheep (9 months) implanted with CP cylinders without BMP (control; **A**; magnified detail in **C**) or with low-dose (LD) BMP-2 (**B**; magnified detail in **D**); quantification of the mineralizing surface in fluorescence-labeled, unstained sections of tibial sections implanted with CP cylinders without (control) or with BMP-2 (**E**; pooled samples from all groups; *n* = 5) or GDF-5 (**F**; pooled samples from all groups; *n* = 5); ^$$$^*p* ≤ 0.005 vs. control. ^++^*p* ≤ 0.01 vs. BMP-2; HA/TCP hydroxyapatite/tricalcium-phosphate

In representative histology images of tibia samples containing CP implants without BMP-2 (Fig. 9A, C) or with BMP-2 (Fig. 9B, D), there was considerable resorption of the CP cylinders, but also new bone formation inside the cylinders (Fig. 9C, D) and in the adjacent corticalis and bone marrow, with a more enhanced bone formation inside and outside the BMP-2 coated CP cylinder (Fig. 9B, D).

When pooling all samples from the BMP-2 and GDF-5 groups (independently of time point and dose), the bone formation parameter mineralizing surface/bone surface was substantially (3.5-fold) and significantly higher than in the controls only in the bone marrow of the BMP-2-coated cylinders, but not in the corticalis (Fig. 9E). This bone formation was also significantly higher than that in the respective GDF-5 group (Fig. 9F).

### Micro-computed tomography (micro-CT)

**Fig. 10:**
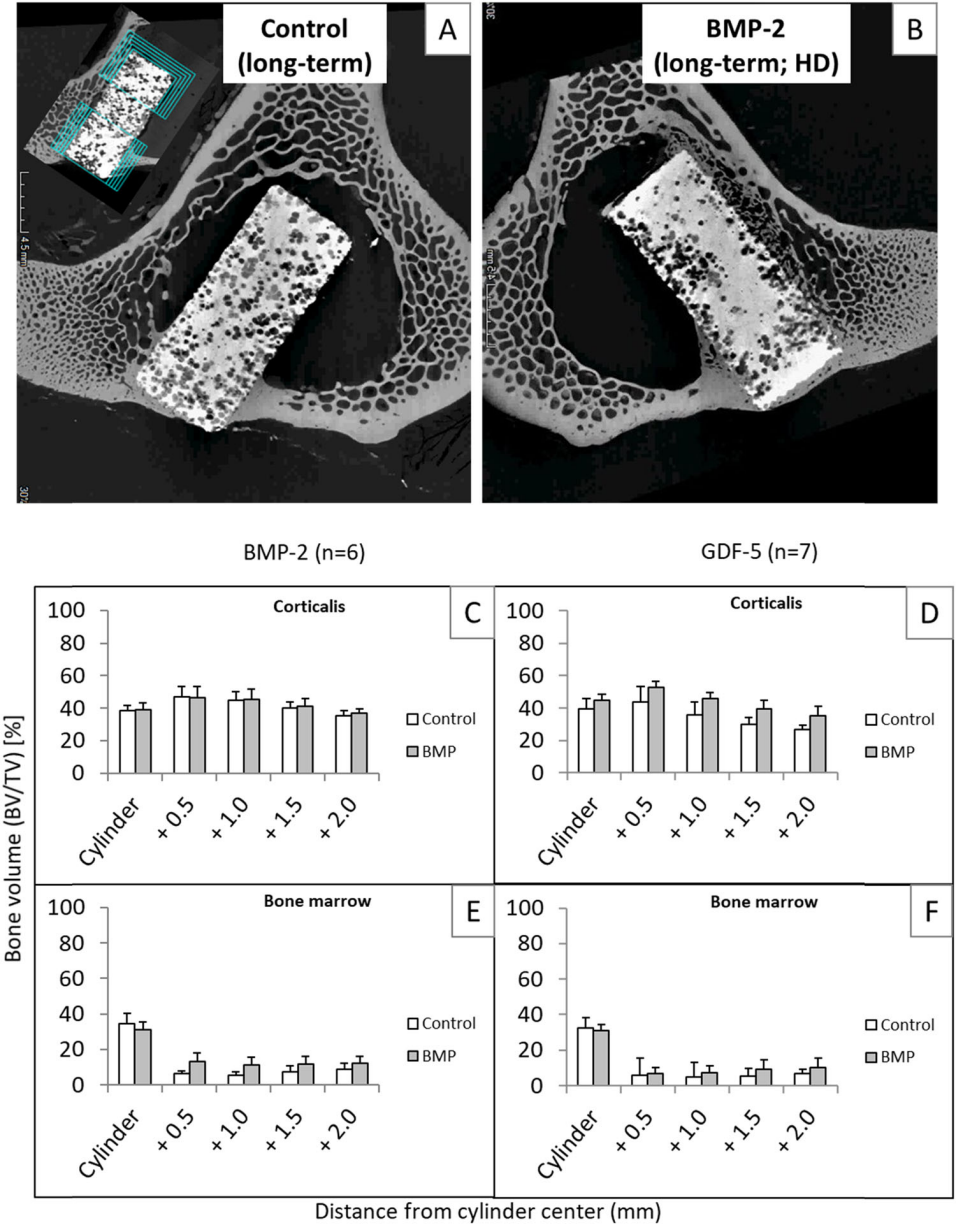
Quantitative evaluation of the micro-computed tomography (micro-CT) of tibial samples from long-term sheep (9 months) implanted with CP cylinders without BMP-2 (control; **A**) or with high-dose (HD) BMP-2 (**B**); the insert in (**A**) shows the separate determination of the bone volume/total volume BV/TV in corticalis and bone marrow in the vicinity of the implant cylinders by application of onion-shell-like cylinders with a radius of 4.0 (cylinder), 4.5, 5.0, 5.5, and 6.0 mm (BMP-2; **C**, **E**; pooled samples from all groups, independent of time point and dose; *n* = 6; GDF-5; **D**, **F**; pooled samples from all groups; independent of time point and dose; *n* = 7)

Representative 9-month micro-CT images confirmed the bone formation in the corticalis and the bone marrow adjacent to the CP cylinders without BMP-2 (Fig. 10A) or with BMP-2 (Fig. 10B), again with a more enhanced bone formation outside of the BMP-2-coated CP cylinder (Fig. 10B).

In pooled samples from the BMP-2 and GDF-5 groups (independently of time point and dose), the predominance of the osteoinductive effects of BMP-2 or GDF-5 coated CP implants in the bone marrow was confirmed (max. 1.95-fold and 1.73-fold induction, respectively), although without significant differences between control and verum groups or between BMP-2 and GDF-5 (Fig. 10C–F).

## Discussion

The present study showed that BMP-2 (and partially GDF-5) significantly increased the bone formation in the vicinity of HA/TCP/DCPD cylinders employed for the filling of defined tibial bone defects in senile, osteopenic sheep [45], a model particularly designed to address the problematic fracture healing in this region. BMP-coated bone replacement materials may therefore be suitable for the therapy of critical size, non-load-bearing bone defects, for example in failed fracture or defect healing.

X-ray evaluation showed that the bone formation significantly increased in both osteoconductive control groups (CP cylinders without BMPs) and osteoinductive verum groups (cylinders with BMPs) until 3 months after implantation. Whereas bone formation in the controls showed a further, mostly significant increase until 6 months, the bone formation in the verum groups reached its peak already at 3 months and thereafter plateaued or decreased. This indicates that the addition of BMPs (in this location in particular BMP-2) both significantly accelerated and augmented the bone formation around the implant cylinder in the tibial defect. These results are in agreement with the osteoinductive effects of BMP-2 or GDF-5 in other bone healing situations, for example dental and facial implants or defects [29, 30, 35, 39, 40], peripheral bone fractures or defects [14, 15, 21–23, 31, 32, 41, 42], and spinal surgery [24–26, 33, 34].

In the X-ray results, the bone formation induced by the BMP-2- or GDF-5-coated CP cylinders reached a plateau already at 3 months after implantation, without any further significant increase until the 9-month time point, although the osteodensitometry indicated a long-term increase in some groups. This suggests on one hand a rapid release of bioactive BMPs from the coated surface of the CP cylinders. Indeed, a very rapid burst release of BMPs in serum has been previously observed from surface-coated HA particles [49] or from other surface-coated bone replacement materials [57–63]. On the other hand, this burst release is followed by an only gradual, but steady further liberation of the BMPs [49], indicating that the sustained release of the BMPs from the CP cylinders in the present study may also be very limited. Although more detailed studies are required to directly analyze the in vivo release from bone implants and thus the relative contribution of initial and long-term release to the observed bone formation, an initially substantial local release of BMPs may be a sufficient trigger for the long-term stimulation of osteogenesis.

Some of the present results indicated a dose-dependency of the BMP-2 effects, as shown by X-ray analyses in pooled 3 month data from short-term and long-term groups (*n* = 12 each; ~2-fold increase by the higher dose) and by osteodensitometry in the long-term BMP-2 groups. This suggests that a BMP-2 dose of ~250 µg may be sufficient for long-term induction of bone formation in the present system, close to the 100 µg recommended for BMP-2, GDF-5 and its mutant BB-1 in the sheep defect model of lumbar osteopenia [24–26]. This dose would be considerably, i.e., 8 and 160 times, lower than previously applied clinical doses of BMP-2 or GDF-5 and would possibly result in a favorable safety profile, even though systematic safety studies are required.

When directly comparing the best-matching dosages of BMP-2 and GDF-5 (250 and 125 µg, respectively), BMP-2 proved more effective than GDF-5 for the induction of bone formation, as shown by X-ray, osteodensitometry, and (independent of the dose) dynamic histomorphometry. This agrees with an early report on a lower biological activity of GDF-5 compared to BMP-2 [64], which also led to the current design of the BMP-2 and GDF-5 doses, and with previous studies describing BMP-2 as the gold standard for its osteoinductive properties in bone replacement/defect surgery [15, 64–66]. However, in own studies in a senile sheep defect model of lumbar osteopenia, matched doses of GDF-5 (1, 5, 100, and 500 µg) proved at least as effective as the respective BMP-2 doses, showing that, possibly depending on the location, GDF-5 or its mutant BB-1 can be as potently osteoinductive as BMP-2 [24–26]. In addition, HA particles coated with 5 µg GDF-5 were more effective than those coated with 10 µg BMP-2 [49]). Although local release of the two BMPs in any of these different bone sites may be different, GDF-5 or its mutant BB-1 may thus still represent a valid option for bone replacement surgery [17, 24, 26, 67–70].

The present study was not a safety study and thus not suitable for a systematic assessment of the safety profile of the BMP-coated CP cylinders. Within the limits of the study design, however, there were no adverse effects previously reported after the spinal surgery of high-dose BMP-2 (as high as 1.95–40 mg; [71]); or after the clinical application of medium dose GDF-5 (0.25–2 mg; [24] and references therein). In addition, there were no signs of local inflammatory infiltration after implantation of the BMP-loaded CP cylinders at any time point, indicating that the BMP or CP components have only negligible pro-inflammatory effects.

Osteoinduction by BMP-2 was most pronounced in the bone marrow adjacent to the CP cylinders, as shown by X-ray, dynamic histomorphometry, and micro-CT. This agrees with the notion that bone marrow of adult animals still contains high numbers of mesenchymal stem cells, which can be locally stimulated by BMP-2 to undergo differentiation into osteoblasts and initiate bone formation [72–75], despite an expectedly decreased proliferation and differentiation potential of such cells in the current aged osteopenic sheep [46]. It thus underlines the enhancing role of BMP-2 for bone defect healing via the recruitment of precursor cells and parallels results showing the induction of bone formation in the vicinity of BMP-2-containing CPC or BMP-2-coated HA particles in lumbar vertebral defects [25, 49]. Since bone defects were generated in both the ipsilateral right (CP cylinder without BMP) and the contralateral left tibia (CP cylinder with BMP) of the same sheep, both defects should have received equal amounts of the oxytetracycline injected for dynamic histomorphometry. Although potential effects of the oxytetracycline on osteogenesis cannot be completely excluded [49], differential effects of the injected oxytetracycline on the two different types of CP cylinders are thus also highly unlikely.

The micro-CT results indicated that the present BMP effects may not be limited to the immediate vicinity of the CP cylinders, but may extend to distances as far as 2 mm from the cylinder. Such a “remote” impact has been previously observed for the osteoconductive effects of CPC and/or CPC with fibers [76], and the osteoinductive effects of BMP-2-containing CPC ([25]; both 2.5 mm) or BMP-2-coated HA particles in lumbar defects of aged osteopenic sheep ([49]; up to 10 mm). Despite the notion that, due to their short half-life and the potential risk of side effects, BMPs cannot be systemically injected but must be used locally and in a carrier-associated form, locally released growth factors may thus be applicable and effective over longer distances than previously thought [71, 77–81].

The in vivo resorbability of the different CP components may have also played a role for the bone formation. In fact, the initial porosity of the HA/TCP implant cylinders (60–80%) with interconnecting pores (diameter 200–500 μm) was regarded as favorable for the ingrowth of blood vessels and bone-forming cells, possibly augmented by the filling/coating of the pores with DCPD, a rapidly resorbable and strongly osteoconductive CP. The histology images supported this assumption, since the CP cylinders in the long-term groups showed non-resorbed regions of HA/TCP, but also pores in which the DCPD had been replaced by newly built bone (Fig. 9C, D; unpublished results).

## Conclusions

BMP-2 (and partially GDF-5) significantly accelerated and augmented the bone formation close to the HA/TCP/DCPD cylinders used to fill defined bone defects in the tibial head of senile, osteopenic sheep. This bone formation reached an early plateau already at 3 months after implantation, with a dose-dependency suggesting that a BMP-2 dose of ~250 µg may be sufficient for long-term induction of bone formation in the present system. This dose is considerably lower (i.e., 8 and 160 times) than previously applied clinical doses of BMP-2 or GDF-5 and may thus result in a favorable safety profile. Indeed, there were no signs of adverse effects or local inflammatory infiltration after the implantation of the BMP-loaded CP cylinders. Thus, BMP-coated CP bone replacement materials may be well-tolerated and suitable for the surgical therapy of critical size, non-load-bearing bone defects in cases of failed fracture or defect healing.
